# Genome-wide linkage analysis of congenital heart defects using MOD score analysis identifies two novel loci

**DOI:** 10.1186/1471-2156-14-44

**Published:** 2013-05-24

**Authors:** Antònia Flaquer, Clemens Baumbach, Estefania Piñero, Fernando García Algas, María Angeles de la Fuente Sanchez, Jordi Rosell, Jorge Toquero, Luis Alonso-Pulpon, Pablo Garcia-Pavia, Konstantin Strauch, Damian Heine-Suñer

**Affiliations:** 1 Institute of Medical Informatics, Biometry, and Epidemiology, Chair of Genetic Epidemiology, Ludwig-Maximilians-Universität, Munich, Germany; 2 Institute of Genetic Epidemiology, Helmholtz Zentrum München, German Research Center for Environmental Health, Neuherberg, Germany; 3 Research Unit, Son Espases University Hospital, Palma de Mallorca, Spain; 4 Pediatric Cardiology, Son Espases University Hospital, Palma de Mallorca, Spain; 5 Genetic Section, Son Espases University Hospital, Palma de Mallorca, Spain; 6 Cardiomyopathy Unit, Department of Cardiology, Puerta de Hierro University Hospital, Madrid, Spain

**Keywords:** Congenital heart defects, Genetics, Linkage analysis, Genome-wide study

## Abstract

**Background:**

Congenital heart defects (CHD) is the most common cause of death from a congenital structure abnormality in newborns and is often associated with fetal loss. There are many types of CHD. Human genetic studies have identified genes that are responsible for the inheritance of a particular type of CHD and for some types of CHD previously thought to be sporadic. However, occasionally different members of the same family might have anatomically distinct defects — for instance, one member with atrial septal defect, one with tetralogy of Fallot, and one with ventricular septal defect. Our objective is to identify susceptibility loci for CHD in families affected by distinct defects. The occurrence of these apparently discordant clinical phenotypes within one family might hint at a genetic framework common to most types of CHD.

**Results:**

We performed a genome-wide linkage analysis using MOD score analysis in families with diverse CHD. Significant linkage was obtained in two regions, at chromosome 15 (15q26.3, *P*_empirical_ = 0.0004) and at chromosome 18 (18q21.2, *P*_empirical_ = 0.0005).

**Conclusions:**

In these two novel regions four candidate genes are located: S*ELS, SNRPA1,* and *PCSK6* on 15q26.3, and *TCF4* on 18q21.2. The new loci reported here have not previously been described in connection with CHD. Although further studies in other cohorts are needed to confirm these findings, the results presented here together with recent insight into how the heart normally develops will improve the understanding of CHD.

## Background

Congenital heart defects (CHD) refer to abnormalities in the heart structure or function that arise at the fetal stages and affect approximately 1% of newborns [1]. Multiple surgeries are almost always required to correct many of the anatomical defects, and quality of life is often greatly compromised. There are many types of CHD. Examples include transposition of the great arteries/vessels (TGA/TGV), tetralogy of Fallot (TOF), double outlet right ventricle (DORV), atrial septal defects (ASD), ventricular septal defects (VSD), bicuspid aortic valve (BAV), and Ebstein’s anomaly, among many others.

Normal heart development involves many regulatory pathways including receptor-ligand interactions (JAGGED/NOTCH*,* TGFB-BMP/TGFBR, VEGF/FLT1-FLK1, NODAL/ACVRA-ACVRB and RTK/RAS), signal transduction (kinases or phosphatases such as MAPK, ERK1/2, calcineurin, or GSK), and transcription factors that determine the expression of cardio-specific genes (protein families with a T-box domain *TBX1, TBX5, and TBX20*, the GATA family *GATA4* and *FOG2* or homeobox domain *NKX2.5* and *NKX2.6*). Mutations that show a greater penetrance and that therefore approximate a monogenic inheritance are those affecting transcription factors or genes transcribed by them [2].

Although the major underlying defects that cause CHD are thought to be mutations in regulators of heart development during embryogenesis, epidemiological data also indicate an environmental influence [2-4]. However, these epidemiological studies mostly suggest risk factors rather than underlying disease mechanisms. Genetic factors for some of the CHD include Mendelian mutations, copy number variants, translocations, and single nucleotide polymorphisms (SNPs) [5-7].

A genetic component of CHD diseases was initially implicated by recurrence in families, and by studies showing a co-segregation of CHD with the deletion 22q11.2 [8]. It has also been shown that individuals with an affected parent are at twofold greater risk, which is even greater if siblings are affected [9]. While many studies have investigated the role of genes in the etiology of inherited and sporadic CHD [10-14] these studies have been focusing on specific cardiac lesions separately. Recently, rare variants have also been reported to be associated with CHD [15]. These rare variants are usually present in less than 1% of the normal population but are overrepresented in selected patients. They are typically inherited from an asymptomatic parent and are therefore not believed to be a sufficient cause of CHD in these patients; other mutations need to be present to lead to CHD.

However, occasionally, different members of one family are observed to suffer from anatomically distinct defects — for example, one member with ASD, one with TOF, and one with VSD. These apparently discordant clinical phenotypes arising within one family are difficult to rationalize. Benson *et al.*[16] observed a coinheritance of different *NKX2.5* mutations, i.e., compound heterozygotes, in members of families with ASD, VSD, and cardiac conduction abnormalities, suggesting that mutations in the same gene can affect different parts of the heart, and therefore cause different types of CHD. McGregor *et al.*[17] reported a statistically significant linkage evidence for a locus on chromosome 14 (within the *HOMEZ* gene) in South Indian cases born to consanguineous parents. However, the linkage finding was not robust in a genetic association follow-up study in a general United States population. Despite the successes over the past few years, much remains unknown about the genetics of CHD. It is believed that much of the missing heritability is likely to be due to rare variants with larger effect sizes as well as epigenetic effects. Their systematic characterization is beyond the means of studies that currently rely on linkage disequilibrium (LD) patterns such as genome-wide association analysis. In such situations taking family information into consideration using a direct mapping approach complemented by segregation techniques will be a useful strategy. For complex diseases linkage analysis is a powerful method for detecting gene effects [18,19]. Especially when looking at rare variants, linkage has the advantage over association of not being prone to allelic heterogeneity. That is, the combination of many weak association signals obtained in a certain region for single variants by means of collapsing methods is automatically performed in the context of linkage analysis.

In this study we performed a detailed genome-wide linkage analysis using MOD scores and 6 families affected by different CHD to test for a common genetic background among different types of heart defects.

## Results and discussion

**Figure 1:**
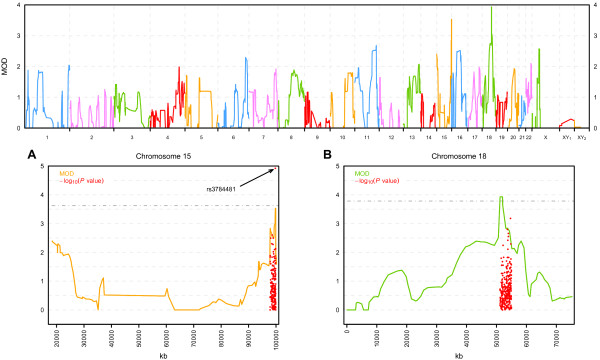
**Genome-wide results for the MOD score analyses.** XY_1_ and XY_2_ represent pseudoautosomal regions 1 and 2, respectively. Figures 1**A** and 1**B** illustrate the MOD score results on chromosome 15 and chromosome 18, respectively. In addition, the –log10(*P* value) of the family-based association test in regions with significant MOD scores are shown as red dots.

The genome-wide results for MOD scores are plotted in Figure 1. No significant results were obtained when modeling parent-specific heterozygote penetrances, resulting in no evidence of parental genomic imprinting in our set of pedigrees. The best results were obtained on chromosome 15 and 18. The results for these two chromosomes are plotted in Figures 1A and 1B, respectively. The highest scores (MOD = 3.9) were detected on chromosome 18 (18q21.2-18q21.3; 51197–52023 kb) comprising 57 SNPs with *P*_empirical_ = (0.0005-0.0006), where *P*_empirical_ refers to the *P* value obtained after simulations were performed. The associated parametric model is a recessive mode of inheritance with incomplete penetrance with a probability of 0.27 to be affected for individuals carrying two copies of the disease allele. Incomplete penetrance refers to the failure of individuals who carry the high risk genotype to exhibit the trait. This phenomenon is often observed in complex diseases. Besides different ways of gene action or degrees of expression, incomplete penetrance can arise from further genetic or environmental factors. The second significant region was at chromosome 15 (15q26.3; 99749–99883 kb) comprising 27 SNPs, all of them with a MOD score of 3.5 and *P*_empirical_ = (0.0004-0.0005). The best-fitting model points to a recessive mode of inheritance, with a probability of 0.98 to be affected for individuals carrying two copies of the disease allele. It should be noted that the estimate of the disease allele frequency obtained by a MOD-score analysis has the largest variance of all trait-model parameters. Furthermore, in some cases, the estimated disease allele frequency will be markedly higher than the true value. This is due to the fact that specifying a higher disease allele frequency can compensate for a general model misspecification and hence lead to robustness in a multipoint analysis [20].

To corroborate these findings, family-based association analysis was performed in these two significant regions with SNPs displaying a MOD >2.5 (*P* value ≤ 0.01). Using this criterion a total number of 304 SNPs (P values between 5 × 10^-04^ and 1 × 10^-02^) and 211 SNPs (*P* values between 4 × 10^-04^ and 4 × 10^-03^) were included in the association analysis for 18q21.2-18q21.3 and 15q26.3, respectively. A total number of 34 SNPs resulted to be significant at a type I error rate of 0.05 on chromosome 18. Nevertheless, none of them was significant after using the Bonferroni correction to adjust for multiple testing. On the other hand, on chromosome 15 a total number of 39 SNPs were found to be nominally significant and only one (rs3784481 at 99749 kb) was significant after Bonferroni correction (*P* value = 0.0025).

To see if one of the two linkage regions may be the causal genetic factor for a particular family we performed parametric linkage (LOD score) analysis of individual pedigrees under the best fitted model for the two linked regions. As shown in Table 1, all the pedigrees contribute positively to the LOD score in both regions (except pedigree 5, chr15). These positive contributions indicate that all types of CHD present in our pedigrees are linked to both regions, supporting the initial hypothesis of this study of a common genetic background for diverse CHD. The negative contribution of pedigree 5 to the LOD score on chromosome 15 has to be treated carefully. Due to the small number of meioses in this pedigree there is a lack of power to detect linkage, and no conclusion should be drawn when analyzing this pedigree individually.

**Table 1:** Parametric LOD scores for each family separately

**Family**	**Heart defect**	**Chr15**	**Chr18**
		**15q26.3**	**18q21.2-18q21.3**
1	VSD	0.615	0.632
2	TOF,TGV	0.324	0.299
3	TOF	0.545	0.602
4	ASD	1.670	1.297
5	VSD,DORV	−0.307	0.602
6	ASD	0.681	0.496
**All**	**VSD,TOF,TGV,ASD,DORV**	**3.529**	**3.929**

When looking in detail at the region around the significant SNPs on chromosome 15, three genes are found very closely to each other (*SELS, SNRPA1*, and *PCSK6*). In addition, one gene is located in the linkage region identified on chromosome 18 (*TCF4*).

### One gene at 18q21.2: TCF4

The potentially relevant *TCF4* gene is located at the linkage peak of chromosome 18*.* This gene encodes transcription factor 4, a basic helix-loop-helix transcription factor. To date, no connection between this gene and CHD has been established. However, it is interesting to note that fairly recently it has been found that in mice the *tcf4* gene is strongly expressed in connective tissue fibroblast. It was demonstrated that connective tissue fibroblasts and *tcf4* critically regulate two aspects of myogenesis: muscle fiber type development and maturation [21]. Taken together this finding in mice and our results could suggest that malfunction of the *TCF4* gene may produce a malformation of the heart muscle tissue which in turn predisposes to a higher risk to develop some type of CHD.

### Three genes at 15q26.3: SELS, SNRPA1, and PCSK6

The *SELS* gene encodes a selenoprotein, which contains a selenocysteine residue at its active site. Studies suggest that this protein may regulate cytokine production, and thus play a key role in the control of the inflammatory response decreasing pro-inflammatory cytokines [22]. Production of cytokines is one of the major mechanisms employed by T-cells to coordinate immune responses. It has been demonstrated that the developing immune system of the neonate not only differs significantly from that of an adult, but also varies based on gestational age [23]. A malfunction of the *SELS* gene might provoke deficient cytokine production by neonatal lymphocytes, and somehow predispose the newborn to a higher risk to develop some CHD. It was also shown that *SELS* is secreted from hepatoma cells and that it associates with low-density lipoprotein particles [24] suggesting also a role in lipid metabolism. Lundell *et al.*[25] had already shown in their study that lipid metabolism was disturbed in infants with CHD. Furthermore, genetic studies in relation to cardiovascular disease have revealed that variation in the *SELS* locus is associated with coronary heart disease and ischemic stroke in women [26].

The *SNRPA1* gene encodes the U2 small ribonucleoprotein A’ (U2 snRNP A’). The action of snRNPs is essential to the removal of introns from pre-mRNA, a critical aspect of post-transcriptional modification of RNA. To date no association has been reported between *SNRPA1* and heart defects. However, post-transcriptional modification is one of the multiple mechanisms that regulate the level and activity of transcription factors which are responsible for regulating formation and function of the heart. Perturbations of transcription factor expression and regulation disrupt normal heart structure and function [27].

The protein encoded by the *PCSK6* gene belongs to the subtilisin-like proproteinconvertase family. The members of this family are proproteinconvertases that process latent precursor proteins into their biologically active products. This encoded protein is a calcium-dependent serine endoprotease that can cleave precursor protein at their paired basic amino acid processing sites. Some of its substrates are transforming growth factor beta related proteins, proalbumin, and von Willebrand factor. In 1998, a study reported abnormalities of von Willebrand factor in adults with CHD [28]. Interestingly, a protein from the same family, proproteinconvertasesubtilisin-like/kexin type 9, which is encoded by the *PCSK9* gene, is a key regulator of plasma low density lipoprotein cholesterol and has emerged as a promising target for prevention and treatment of coronary heart diseases. A recent genome-wide association study further bolstered the importance of *PCSK9* by establishing a link between a nearby SNP and early onset myocardial infarction [29].

## Conclusions

We have conducted a whole genome linkage analysis for CHD using MOD score analysis. We used a total number of 177,568 SNPs providing a good coverage of the genome in a sample of 6 families of Spanish origin with members affected with anatomically distinct CHD: ASD, VSD, TOF, TGV, and DORV. The aim of this study was the identification of chromosomal regions that are implicated in a possible common genetic framework shared by distinct types of CHD. Two interesting linkage regions were found, on chromosome 15 and 18. The highest linkage peak was obtained at position 18q21.2 (LOD = 3.9, *P*_empirical_ = 0.0005) and the second highest peak at 15q26.3 (LOD = 3.5, *P*_empirical_ = 0.0004) using MOD-score analysis. In addition, at 15q26.3 a significant association was obtained for a SNP (rs3784481, *P* value = 0.0025) using family-based association analysis. Imprinting analysis of the data in the present study did not confirm the hypothesis of a possibly imprinted locus. When looking at these two linkage regions in detail, four genes are located there, three very close to each other at 15q26.3 and one at 18q21.2.

As in all research work, our results have some unavoidable limitations. They have been obtained using linkage techniques with a small sample size. It is well known that large, multi-generation pedigrees are the most informative for linkage analysis. However, such pedigrees are difficult to find when investigating complex diseases with a high and early mortality such as CHD. That might explain why to date there have been so few genome-wide linkage scans involving affected families with CHD. Therefore, despite the fact that our study comprising 6 families is small compared with linkage studies for other disorders, it is, thus far, the largest family study performed in CHD. Moreover, our findings on chromosome 15 are corroborated by family-based association analysis (with 7 additional trios). Clearly, recruiting more families with CHD will help to verify this linkage evidence as well as to identify new linkage regions.

We report in this study two genetic regions linked to CHD predisposition. There are three candidate genes located in the 15q26.3 region very close to each other (*SELS*, *SNRPA1* and *PCSK6*) and one gene at 18q21.2 (*TCF4).* Only one of these genes, *SELS,* was previously found to be implicated in cardiovascular disorders. The other three genes reported in this study may be considered as novel candidate loci for CHD.

While several genome scans have reported susceptibility genes for specific types of CHD, very few (only one [17], and the one presented here) have identified susceptibility genes that predispose to developing anatomically distinct types of CHD and should form the basis of new analyses of both linkage and association studies on CHD. Future linkage studies may benefit in power from larger sample size, and also case–control studies will benefit from direction or corroboration given by linkage scans. For example, the extensive multiple testing problem in genome-wide association studies can be partially alleviated by a weighted Bonferroni correction or Bayesian analysis that employs linkage data [30]. The results presented here should facilitate the discovery of both common and rare variants that confer genetic susceptibility for CHD and help the understanding of heart mechanisms. Understanding the etiology of CHD will also help to identify possible complications and risk factors for surgery or treatment, as patients (babies and children) with genetic syndromes or extracardiac anomalies are generally at higher risk of operative mortality and morbidity.

## Methods

### Subjects

**Figure 2:**
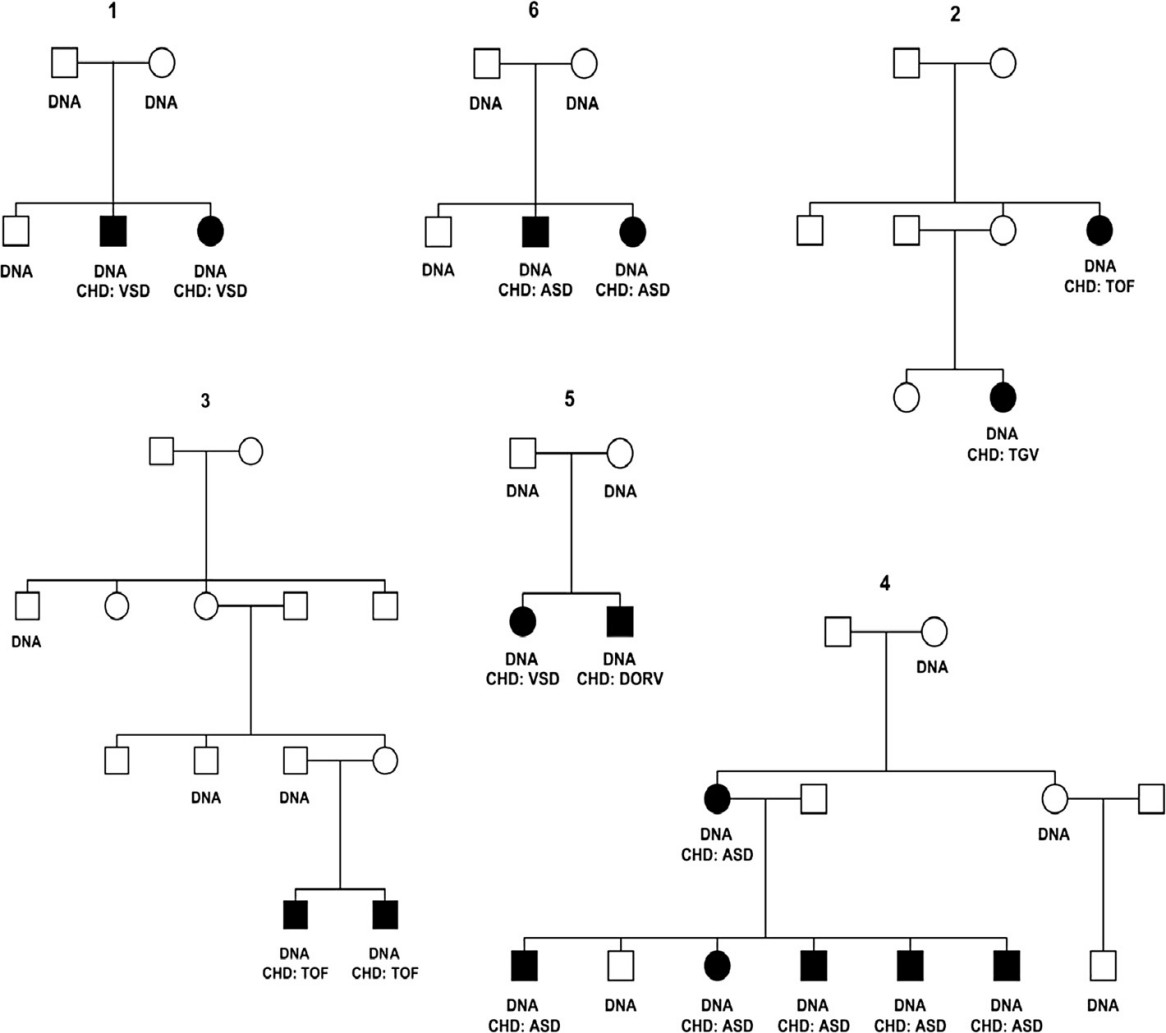
**Pedigree structures.** Pedigree structures used to perform linkage analysis. In addition, 7 trios (an affected proband and both parents) affected with CHD - DORV, VSD, TOF, TGV, or ASD – were used in family-based association analysis.

A total number of 6 families comprising 48 individuals (19 females, 29 males) and 7 trios (an affected proband and both parents) of Spanish origin were recruited at the University Hospital Son Espases (HUSE) of the Balearic Islands and at the Puerta del Hierro University Hospital in Madrid. Pedigree structures are illustrated in Figure 2. The inclusion criterion for all patients was a CHD present at birth. Patients and their families were asked to participate in the study within the clinical practice and blood samples were taken. All members included in the study were checked for the presence of deletions or duplications using the same SNP-array analysis. Individuals that were found to carry a suggestive or clearly causative deletion or duplication (22q11.2 or other) were excluded from the analysis. All participants provided their written informed consent to participate in the study. A statement of informed consent form was signed by the parents of all participating children under 18 years old, following protocols approved by the ethical committee for clinical research of the Balearic Islands (IB 963/08; Comité ético de investigación clínica de las Islas Baleares; CEIC). The study conformed to all ethical guidelines of the institutions involved. Research at the HUSE and Puerta del Hierro follow the guidelines included in the June 1964 Declaration of Helsinki (entitled “Ethical Principles for Medical Research Involving Human Subjects”). All sample identifiers were re-encoded in a way that only the clinicians are able to access personal information.

### DNA isolation and genotyping

All patients had a single tube of blood drawn at the time of enrollment, which was collected centrally for subsequent DNA extraction. Genotyping reactions of 52 individuals were performed at the Spanish genotyping center (CeGen) facilities in Barcelona using the Illumina (California, USA) Human660W-Quad chip, following the manufacturer’s instructions.

### Data quality control

Because genotyping errors can lead to incorrect inferences about gene flow in pedigrees and may reduce the effectiveness of linkage analysis, strict quality controls (QC) were performed in our data. First of all, the software Graphical Representation of Relationship Errors (GRR) was used to identify errors in the structure of the pedigrees and to eliminate duplicate samples, monozygotic twins, and unrelated subjects [31]. In one family GRR detected an inconsistency involving a mother and her two children. Eventually it turned out that the children’s genotypes were mutually consistent and only the mother’s genotypes were removed from the analysis. All individuals were also tested for sex consistency. Afterwards, the PEDCHECK software was used to detect marker typing incompatibilities [32]. A total number of 3271 (0.6%) incompatibilities were detected and those genotypes were removed from the data. Additional QC was undertaken to check for unlikely double recombinants. Two or more close recombination events on the same chromosome are uncommon due to the chance distribution of chiasmata, and because of interference. To detect such problematic genotypes the “error detection” option from the MERLIN v1.1.2 software was used and the unlikely genotypes were subsequently removed from the data using the “pedwipe” option [33]. Once Mendelian inconsistencies and unlikely genotypes were resolved, SNPs with call rates below 95%, monomorphic SNPs, and SNPs with a missing genetic position were also removed from the analysis. At the end, 93.95% of the original SNPs survived the QC. Allele frequencies were then estimated with MERLIN v1.1.2 using a maximum-likelihood procedure.

### Data preparation for linkage analysis

In linkage, when analyzing SNPs in pedigrees where some parental genotypes are missing it is necessary to model for marker-marker LD prior to the analysis. Ignoring such LD can result in severe biases in linkage results [34]. That is why pairwise LD between each pair of SNP loci was analyzed, using the procedure ldmax in the GOLD software [35]. ldmax calculates r^2^ values from haplotype frequencies estimated via the expectation maximization (EM) algorithm of Excoffier and Slatkin [36]. In our data, whenever a pair of SNPs was in LD (r^2^ > 0.20), one of the two SNPs was removed from the analysis. With the resulting data set a genome-wide linkage analysis was performed on a total number of 6 pedigrees with sizes varying from 4 to 13 members. After QC, a total number of 177,568 SNPs was analyzed, with 174,475 from the autosomes, 3078 from the X-chromosome, and 15 SNPs from the pseudoautosomal regions (PAR1 and PAR2).

### Statistical methods

In parametric linkage analysis (LOD scores) the trait-model parameters, i.e., the penetrances and disease allele frequency, must be specified prior to the analysis. It is well known that the power to detect linkage decreases when the specified model is not sufficiently close to the true one. Consequently, we calculated multipoint MOD scores, i.e., LOD scores that were maximized over trait-model parameters using the program GENEHUNTER-MODSCORE (GH-MOD v3.0) [37]. This program calculates MOD scores automatically by varying the disease-allele frequency and three penetrances. We used the option “modcalc single” to perform a separate maximization for each genetic position assumed for the putative disease locus. This procedure yields a MOD score in conjunction with the penetrances and disease allele frequency of the best-fitting trait model for every genetic position. MOD score analysis represents a comprehensive way to analyze linkage data when the mode of inheritance is unknown; in general this procedure is particularly well suited for the genetic dissection of a complex trait [38].

It has also been observed that the CHD risk for children of mothers with a CHD is consistently higher than for those of fathers with a CHD [39,40]. One possible explanation for the increased transmission through females is a paternal imprinting mode of inheritance, i.e., offspring will be affected only if the inherited disease allele comes from the mother. Hence, we also checked for a parent-of-origin effect using two different penetrances for heterozygous individuals to take the parent who transmitted the disease allele into account (see “imprinting” option of GH-MOD v3.0).

A MOD-score analysis leads to an inflation of the type I error when compared to LOD scores calculated under a single parametric model. Therefore, in the context of MOD-score analysis, significance criteria for LOD scores cannot be applied without correction. To assess significance for a MOD score empirical *P* values need to be calculated. We used 5000 replicates, each one generated with Monte-Carlo simulations of marker alleles under the assumption of no linkage and Hardy-Weinberg equilibrium, using the same pedigree structure, affection status, marker spacing, and allele frequencies as in the real data set. Replicates were analyzed like the original data. The empirical *P* value was obtained as the proportion of replicates showing a MOD score equal to or higher than the one observed in the real data set.

We computed MOD and MOD-imprinting for all autosomes. The MOD score has not been implemented so far for the X-chromosome. Therefore, we performed LOD-score analysis assuming different genetic models for the X-chromosome, ranging from completely dominant to completely recessive modes of inheritance. In addition, the PAR1 and PAR2 were also investigated. Genetic markers in the PARs follow the same inheritance pattern as autosomal markers, becoming progressively more sex-linked (i.e., recombination events are becoming rarer between X and Y) as they approach the pseudoautosomal boundary. When using multipoint linkage analysis in the PARs, it is crucial to use sex-specific maps since there exists a marked difference in recombination frequencies between males and females [41]. We used pseudoautosomal sex-specific genetic coordinates [41] implemented in the Rutgers map [42]. The sex-specific genetic positions were interpolated using a local regression with a quadratic fit as implemented by the “locfit” package [43] of the statistical software R. In order to obtain a mapping that is monotonous in the base pair positions we used R’s “monoProc” package which monotonizes the local regression fit [44].

Finally, only in those regions where the most significant linkage signals were obtained, to corroborate and narrow the significant regions, family-based association analysis together with the trio families was performed using the LAMP software [45]. LAMP uses a maximum likelihood model to extract the exact information on genetic linkage and association from samples of unrelated individuals, sib pairs, trios, and larger pedigrees. *P* values obtained by the family-based association analysis were adjusted using Bonferroni correction (using the total number of SNPs in each specific region of linkage) to account for multiple testing. We used an overall type I error rate of 0.05.

## Competing interests

The authors declare that they have no competing interests.

## Authors’ contributions

AF carried out all statistical analyses, interpretation of the results and wrote the manuscript. CB carried out all the simulation analyses, prepared the figures and edited the manuscript. EP carried out the SNP-array DNA preparation and partial analysis. FGA, MADLF, JT, LAP and PGP characterized clinically heart defects of patients. JR characterized clinical dysmorphology and evaluated inheritance. KS critically revised the manuscript and all statistical content. DHS provided samples and revised the manuscript critically for important molecular content. All authors gave final approval of the version to be published.
